# Huntingtin-Associated Protein 1A Regulates Store-Operated Calcium Entry in Medium Spiny Neurons From Transgenic YAC128 Mice, a Model of Huntington’s Disease

**DOI:** 10.3389/fncel.2018.00381

**Published:** 2018-10-26

**Authors:** Magdalena Czeredys, Vladimir A. Vigont, Vasilisa A. Boeva, Katsuhiko Mikoshiba, Elena V. Kaznacheyeva, Jacek Kuznicki

**Affiliations:** ^1^Laboratory of Neurodegeneration, International Institute of Molecular and Cell Biology in Warsaw (IIMCB), Warsaw, Poland; ^2^Institute of Cytology, Russian Academy of Sciences (RAS), St. Petersburg, Russia; ^3^Laboratory for Developmental Neurobiology, RIKEN Brain Science Institute (BSI), Saitama, Japan

**Keywords:** Huntington’s disease, huntingtin, huntingtin-associated protein 1 isoform A, YAC128, medium spiny neurons, store-operated calcium entry, inositol-(1, 4, 5)triphosphate receptor type 1, tetrahydrocarbazole

## Abstract

Huntington’s disease (HD) is a hereditary neurodegenerative disease that is caused by polyglutamine expansion within the huntingtin (HTT) gene. One of the cellular activities that is dysregulated in HD is store-operated calcium entry (SOCE), a process by which Ca^2+^ release from the endoplasmic reticulum (ER) induces Ca^2+^ influx from the extracellular space. HTT-associated protein-1 (HAP1) is a binding partner of HTT. The aim of the present study was to examine the role of HAP1A protein in regulating SOCE in YAC128 mice, a transgenic model of HD. After Ca^2+^ depletion from the ER by the activation of inositol-(1,4,5)triphosphate receptor type 1 (IP_3_R1), we detected an increase in the activity of SOC channels when HAP1 protein isoform HAP1A was overexpressed in medium spiny neurons (MSNs) from YAC128 mice. A decrease in the activity of SOC channels in YAC128 MSNs was observed when HAP1 protein was silenced. In YAC128 MSNs that overexpressed HAP1A, an increase in activity of IP_3_R1 was detected while the ionomycin-sensitive ER Ca^2+^ pool decreased. 6-Bromo-*N*-(2-phenylethyl)-2,3,4,9-tetrahydro-1*H*-carbazol-1-amine hydrochloride (C_20_H_22_BrClN_2_), identified in our previous studies as a SOCE inhibitor, restored the elevation of SOCE in YAC128 MSN cultures that overexpressed HAP1A. The IP_3_ sponge also restored the elevation of SOCE and increased the release of Ca^2+^ from the ER in YAC128 MSN cultures that overexpressed HAP1A. The overexpression of HAP1A in the human neuroblastoma cell line SK-N-SH (i.e., a cellular model of HD (SK-N-SH HTT138Q)) led to the appearance of a pool of constitutively active SOC channels and an increase in the expression of STIM2 protein. Our results showed that HAP1A causes the activation of SOC channels in HD models by affecting IP_3_R1 activity.

## Introduction

Huntington’s disease (HD) is an autosomal dominant neurodegenerative disorder that is caused by the expansion of a CAG trinucleotide repeat in exon 1 of the huntingtin (*HTT*) gene, which is translated into polyglutamine residues (polyQ) in the HTT protein. The length of the polyglutamine tract normally does not exceed 35 glutamine residues, whereas longer tracts are associated with the development of pathology.

HTT-associated protein-1 (HAP1) was the first HTT binding partner that was identified (Li et al., 1995; Gutekunst et al., 1998; Page et al., 1998). The association between HTT and HAP1 is promoted by the polyQ expansion of mutated Htt (mutHTT; Li et al., 1995, 1998). In rodents, two HAP1 protein isoforms—HAP1A and HAP1B—that differ in their carboxy termini are expressed via alternative splicing. Both isoforms bind HTT (Li et al., 1995; Nasir et al., 1998). Only one HAP1 isoform has been identified in humans and shares higher homology with HAP1A (Li et al., 1998).

HAP1 has several functions under physiological conditions. It is known to play a role in signal transduction, the regulation of vesicular transport, gene transcription and the regulation of membrane receptor recycling (Wu and Zhou, 2009). HAP1 regulates endocytosis and interacts with multiple trafficking-related proteins (Mackenzie et al., 2017). In β-cells, HAP1 helps regulate the transport of insulin-containing secretory granules along cortical actin filaments (Wang et al., 2015). It is also involved in neuronal differentiation (Huang et al., 2015). The expression of HAP1 was previously found in mouse medium spiny neurons (MSNs; Tang et al., 2004), which are affected in the striatum in humans who suffer from HD. In pathological states, HAP1 affects different signaling pathways. For example, it plays a role in downregulating γ-aminobutyric acid-ergic neurotransmission during cerebral ischemia (Mele et al., 2017). In Down syndrome, DYRK1A was found to regulate the Hap1-Dcaf7 interaction and postnatal growth (Xiang et al., 2017).

Alterations of intracellular Ca^2+^ signaling are believed to play a significant role in the pathogenesis of neurodegenerative disorders (Wojda et al., 2008; Bezprozvanny, 2009; Berridge, 2010; Riazantseva et al., 2012; Imamura et al., 2016; Alzheimer’s Association Calcium Hypothesis Workgroup, 2017; Surmeier et al., 2017), including HD (Wu et al., 2011; Czeredys et al., 2017; Kolobkova et al., 2017). One of the most ubiquitous pathways for Ca^2+^ uptake is store-operated calcium entry (SOCE; Poggioli and Putney, 1982; Bouron et al., 2005; Prakriya and Lewis, 2015). The engagement of plasma membrane receptors through G-proteins or the tyrosine kinase cascade leads to the activation of phospholipase C (PLC), which produces inositol(1,4,5)triphosphate (IP_3_) through the cleavage of phosphatidylinositol(4,5)bisphosphate. The consequent activation of IP_3_ receptors (IP_3_Rs) results in the Ca^2+^ release, a decrease in endoplasmic reticulum (ER) Ca^2+^ content and the activation of Ca^2+^ influx through SOC channels (Prakriya and Lewis, 2015). SOCE is also present in neurons (Klejman et al., 2009; Venkiteswaran and Hasan, 2009; Majewski and Kuznicki, 2015; Moccia et al., 2015).

SOCE was previously shown to be elevated in MSNs that were cultured from YAC128 mice, a transgenic mouse model of HD (Wu et al., 2011; Czeredys et al., 2017), and in different cell models of HD that express mutHTT or its N-terminal fragment that contains an expanded polyQ region (Wu et al., 2011; Vigont et al., 2014, 2015; Nekrasov et al., 2016). An increase in SOCE may lead to neurodegeneration that is observed in HD (Wu et al., 2016, 2018). The increase in Ca^2+^ efflux from the ER that is activated by mutHTT, in turn, enhances SOCE from the extracellular space in MSNs from transgenic YAC128 mice (Wu et al., 2011, 2016).

Using a yeast two-hybrid screen, HAP1A was identified as a binding partner of IP_3_R1, which interacts with its C-terminal part (Tang et al., 2003). Furthermore, HAP1A potentiated the effect of mutHTT on IP_3_R1-mediated Ca^2+^ release in mouse MSNs (Tang et al., 2004). This interaction with IP_3_R1 destabilized ER Ca^2+^ content and may cause perturbations in intracellular Ca^2+^ signaling.

Significant upregulation of some members of Ca^2+^ signalosomes was found in the striatum in YAC128 mice (Czeredys et al., 2013). The most significantly upregulated protein in the striatum in the HD mouse model was HAP1 protein. Greater SOC channel activation in HD could thus be explained by an increase in the release of Ca^2+^ from the ER because of the facilitated opening of IP_3_R1 by HAP1 overexpression. Therefore, we tested whether HAP1 overexpression in the striatum in the YAC128 mouse model of HD affects Ca^2+^ homeostasis in MSNs that are cultured from YAC128 mice. We found that HAP1A abnormally increased DHPG-induced SOC by activating IP_3_R1 in YAC128 MSNs that overexpressed HAP1A. This effect was restored by tetrahydrocarbazole, which was previously described by us as a stabilizer of the elevation of SOC in HD (Czeredys et al., 2017). The same restorative action was observed for IP_3_ sponge, an inhibitor of IP_3_R1 (Iwasaki et al., 2002; Uchiyama et al., 2002). In the cellular model of HD (SK-N-SH HTT138Q), we found that HAP1A caused the constitutive activity of SOC channels and increased the expression of STIM2 protein.

## Materials and Methods

### Medium Spiny Neurons

MSN cultures were prepared from embryonic day 18 hemizygous YAC128 (Slow et al., 2003) and wildtype mouse striata. Pregnant YAC128 mice were provided by the Laboratory of Genetically Modified Animals Breeding in the Center of Experimental Medicine (CEM-CePT) in Mossakowski Medical Research Centre, Polish Academy of Sciences (Warsaw, Poland). Animal care was in accordance with the European Communities Council Directive (86/609/EEC). The experimental procedures were approved by the Local Commission for the Ethics of Animal Experimentation no. 1 in Warsaw (approval no. 548/2014 and 658/2015). Brains were removed from mouse embryos and collected in cold Hibernate E (Invitrogen) supplemented with penicillin (100 U/ml)/streptomycin (100 μg/ml; Invitrogen). The tails of the embryos were collected and genotyped by polymerase chain reaction (PCR). The striata were isolated, rinsed three times in cold Hibernate E solution, and treated with 2.5% trypsin for 20 min. The tissue was then rinsed in 37°C Hank’s solution and dissociated by pipetting. The MSNs were plated in Neurobasal medium (Invitrogen) supplemented with 2% B27 (Invitrogen), 0.5 mM glutamine (Sigma) and a penicillin (100 U/ml)/streptomycin (100 μg/ml) mixture. For single-cell Ca^2+^ measurements MSNs were plated at a density of 70,000 cells per glass well in Corning BioCoat Poly-D-Lysine/Laminin Cellware 8-Well Culture Slides (Corning). For real-time PCR and Western blot, MSNs were seeded on a cover glass (25 mm diameter) that was coated with poly-D-lysine (38 μg/ml; Sigma) and laminin (2 μg/ml; Roche) at 400,000 cells/plate. After 2 h, the medium was replaced with the fresh culture medium that contained Neurobasal medium (Invitrogen) supplemented with 2% B27 (Invitrogen), 0.5 mM glutamine (Sigma) and a penicillin (100 U/ml)/streptomycin (100 μg/ml) mixture (Invitrogen). The cultures were maintained at 37°C in a humidified 5% CO_2_/95% air atmosphere.

### Human Neuroblastoma Cells (SK-N-SH)

SK-N-SH cells from the collection of the Institute of Cytology, Russian Academy of Sciences (RAS), were cultured in Dulbecco’s Modified Eagle Medium (DMEM; Biolot) with 5% fetal bovine serum (FBS; Gibco) and 80 g/ml gentamicin (Biolot). Two days before the experiments, the cells were plated onto coverslips (3 × 3 mm) that were coated with 0.01% poly-L-lysine (Sigma) for better adhesion. On the second day, the cells were transiently cotransfected using Lipofectamine as previously described (Wu et al., 2011) with a plasmid that encoded full-length mutant HTT 138Q (Goldberg et al., 1996) and pLenti-C-mGFP or HAP1A-pLenti-C-mGFP expression constructs in a 3:1 molar ratio. The transfected cells were identified by green fluorescent protein (GFP) fluorescence, and SOC currents were measured 24 h after transfection using the patch clamp technique as described below.

### Human Embryonic Kidney Cells (HEK293 T/17)

HEK293 T/17 cells were obtained from the American Type Culture Collection and grown in DMEM (Gibco). All media contained 10% FBS and a penicillin (100 U/ml)/streptomycin (100 μg/ml) mixture (Invitrogen).

### Plasmids

HAP1A or HAP1B mouse cDNA clones in pCMV6-ENTRY that originated from Origene (catalog no. NM010404 or NM177981) were cloned between the *Sgf*I and *Mlu*I sites into pLenti-C-mGFP (catalog no. PS100071, Origene) for HAP1 overexpression. For HAP1 silencing, commercially available short-hairpin RNA (shRNA) sequences in pLenti-GFP (catalog no. TL500930C, Origene) were used. As a control, scrambled shRNA against pLenti-GFP was applied (catalog no. TR30021, Origene). A plasmid with a glutathione *S*-transferase (GST)-tagged IP_3_R1 ligand binding region (226–604 amino acid region) with a point mutation (R441Q: m49) or a negative control plasmid with a point mutation (K508A: m30) was recloned from pEF-GSTm49-IRES-GFP or pEF-GSTm30-IRES-GFP (Iwasaki et al., 2002; Uchiyama et al., 2002). Vectors were digested with *Not*I, followed by Klenow (Thermo Fisher Scientific, Waltham, MA, USA) fragment treatment to obtain blunt ends. Linear vectors were then digested using *Xba*I. GST-tagged IP_3_R1 sponge or a control fragment was cloned between the *Xba*I and *Hinc*II sites into the pUltra-Chili vector. To obtain the GST-tagged IP_3_R1 sponge or control fragment in the frame with the pUbe promoter in the pUltra-Chili plasmid, the *Xba*I restriction site together with the corresponding cytosine on its 3’ end was deleted using PCR with Phusion polymerase (Thermo Fisher Scientific, Waltham, MA, USA) and primers for deletion. For GST-tagged IP_3_R1 sponge, the following primers were used: 5’-TGCCGGATCGGCACCAT-3’ (forward) and 5’-AGGCCCGGGATTCTCCTC-3’ (reverse). For the control GST-tagged IP_3_R1 fragment, the following primers were used: 5’-CGATGCACCATGTCCCCT-3’ (forward) and 5’-AGGCCCGGGATTCTCCTC-3’ (reverse). The DNA ends were phosphorylated using PNK kinase (Thermo Fisher Scientific, Waltham, MA, USA) and ligated using T4 DNA ligase (Thermo Fisher Scientific, Waltham, MA, USA). Cloning was confirmed by both restriction digestion and sequencing.

### Virus Production and Transduction

All gene overexpression was performed by transducing the cells with lentiviruses that carried pLenti-C-mGFP plasmids with HAP1A or HAP1B sequences or pUltra-Chili plasmids with m49-dTomato or m30-dTomato sequences. The knockdown of HAP1 was performed by transducing the cells with lentiviruses that carried pLenti-GFP plasmids that targeted shRNAs against the HAP1 sequence or scrambled shRNA as a control. The viruses were prepared in HEK293 T/17 cells using the Ca^2+^ phosphate transfection method. Supernatants were collected 48–72 h after transfection, filtered through 0.45-μm membranes, and concentrated in Vivaspin 100-kDa units (Sartorius) in a swing-out rotor at 1,000 × *g*. Day *in vitro* 5 (DIV5) MSN cultures were transduced with lentiviruses to overexpress HAP1 isoforms and the IP_3_ sponge inhibitor or to silence HAP1. For pLenti-C-mGFP, shHAP1 in pLenti-GFP, m49-dTomato or m30-dTomato in pUltra-Chili viral infection, efficiency was ~90%. For HAP1A or HAP1B in pLenti-C-mGFP viral infection, efficiency was ~20%. Experiments with MSNs started at least 1 week after virus transduction.

### Gene Expression Analysis

Total RNA from MSNs was isolated with the RNeasy Plus Kit (Qiagen). cDNA was synthesized with random hexamer primers and SuperScript III RNase H-Reverse Transcriptase (Invitrogen). The samples were examined by real-time PCR in a 7900HT Real-Time PCR System (Applied Biosystems). Commercial TaqMan primers and probes (Applied Biosystems) were used to quantify specific mRNA levels: *Gapdh* (control), transient receptor potential channel type 1 (*Trpc1*), *Hap1*, *Itpr1* (*InsP3R1*) and *CacyBP/SIP*. The primers that were used for mRNA that encoded genes that are involved in SOCE (i.e., *Stim2-1*, *Stim2-2*, *Stim1*, *Orai1*, *Orai2* and *Orai3*) were provided by Prof. Barbara Niemeyer (Table 1). For relative quantification, the fold change in expression was quantified by normalizing the threshold cycle (CT) values of the target mRNAs to the CT values of the internal control *Gapdh* in the same samples (ΔCT = CT_Target_ − CT_Gapdh_). It was further normalized to the wildtype control (ΔΔCT = ΔCT − CT_Control_). The fold change in expression was then obtained by calculating 2^∧^-ddCt. The relative mRNA levels of the analyzed genes were measured as 2^∧^-dCt.

**Table 1:** Primers for real-time polymerase chain reaction (PCR) for store-operated calcium entry (SOCE) players.

Gene	Primer	Sequence 5′-3′
mORAI1	for	5′-ATGAGCCTCAACGAGCA-3′
	rev	5′-GTGGGTAGTCATGGTCTG-3′
mORAI2	for	5′-TGGAACTCGTCACGTCTAAC-3′
	rev	5′-GGGTACTGGTACTTGGTCT-3′
mORAI3	for	5′-GTACCGGGAGTTCGTGCA-3′
	rev	5′-GGTATTCATGATCGTTCTCC-3′
mSTIM1	for	5′-CAGAGTCTGCATGACCTTCA-3′
	rev	5′-GCTTCCTGCTTGGCAAGGTT-3′
mSTIM2.1	for	5′-CGAGGTCGCTGCCTCCTATC-3′
	rev	5′-CACGTGGTCAGCTCAGAGAG-3′
mSTIM2.2	for	5′-GGACGAGGCAGAAAAAATTAAAAAG-3′
	rev	5′-CACGTGGTCAGCTCAGAGAG-3′

### Immunoblotting

MSN cultures were extracted in ice-cold RIPA buffer (50 mM Tris (pH 7.5), 150 mM NaCl, 1% NP-40, 0.5% NaDOC, 0.1% sodium dodecyl sulfate (SDS), and 1 mM ethylenediaminetetraacetic acid (EDTA)) that contained mini complete protease inhibitor cocktail (Roche) and phosphatase inhibitors (Sigma). The lysed cells were centrifuged at 12,000× *g* for 10 min. Protein extracts (20 μg) were separated by 10% SDS-polyacrylamide gel electrophoresis (PAGE), transferred to a Protran nitrocellulose membrane (Whatman), and blocked for 2 h at room temperature in TBS-T (50 mM Tris-HCl (pH 7.5), 150 mM NaCl, and 0.1% Tween 20 plus 5% dry nonfat milk). The nitrocellulose sheets were then incubated at 4°C overnight in blocking solution with primary polyclonal antibodies against GST (1:5,000; catalog no. G7781, Sigma) and monoclonal antibodies against HAP1 protein (1:300; catalog no. 611302, BD Transduction Laboratories), CacyBP/SIP (1:1,000; catalog no. ab51288, Abcam), or GFP (1:1,000; catalog no. 11814460001, Roche). As a control, secondary polyclonal antibodies against GAPDH (1:1,000; catalog no. sc-25778, Santa Cruz Biotechnology) or vinculin (1:10,000; catalog no. Ab129002, Abcam) and a monoclonal antibody against β-actin (1:10,000; catalog no. A5441, Sigma) were used, followed by incubation with horseradish peroxidase-conjugated anti-rabbit IgG secondary antibody (1:10,000; catalog no. A0545, Sigma) for 1 h at room temperature. The signal was detected using an enhanced chemiluminescence substrate (Amersham Biosciences).

SK-N-SH cells were grown in 50-mm Petri dishes. After transfection, they were lysed in 10 mM Tris-HCl buffer (pH 7.5) with 150 mM NaCl, 1% Triton X-100, 1% NP40 (Nonidet P40, nonionic detergent nonylphenoxypolyethoxylethanol), 2 mM EDTA, 0.2 mM phenylmethanesulfonylfluoride (PMSF; serine protease inhibitor), and protease inhibitor cocktail (Roche). Proteins were resolved by electrophoresis in 8% polyacrylamide gel and transferred to a PVDF membrane, pretreated with methanol and transfer buffer (48 mM Tris, 39 mM glycine and 5% methanol). The membrane was incubated with 5% milk for 1 h at room temperature and treated with primary polyclonal anti-TRPC1 antibody (1:200; catalog no. ACC-010, Alomone Labs), anti-Orai1 antibody (1:1,000; catalog no. O8264, Sigma), or anti-STIM2 antibody (1:500); catalog no. 4917, Cell Signaling Technology) and peroxidase-conjugated goat anti-rabbit IgG secondary antibody (1:30,000; catalog no. A0545, Sigma). For STIM1 detection, the PVDF membrane was treated with the primary monoclonal anti-STIM1 antibody (1:250; catalog no. 610954, BD Bioscience) and peroxidase-conjugated goat anti-mouse IgG heavy-chain constant region secondary antibody (1:30,000; catalog no. A0168, Sigma). Target proteins were visualized using the Super Signal Chemiluminescent Substrate (Pierce). All of the experiments were performed in at least three replications with different cell lysates. Monoclonal anti-α-tubulin antibody (1:1,000; catalog no. T6074) was used as the loading control. Relative protein content was estimated using standard software for comparing the intensity of bands in the scanned blots.

### Application of Tetrahydrocarbazole

The tetrahydrocarbazole 6-bromo-*N*-(2-phenylethyl)-2,3,4,9-tetrahydro-1*H*-carbazol-1-amine hydrochloride (C_20_H_22_BrClN_2_; catalog no. 5265927, ChemBridge), which stabilizes the increase in Ca^2+^ efflux from the ER in HEK293-PS1 cells (Honarnejad et al., 2013) and reduces SOC channel activity in a model of HD (Czeredys et al., 2017), was used. The compound was added to each well at a final concentration of 10 μM in 0.02% dimethyl sulfoxide (DMSO). As a control, MSN cultures were treated with 0.02% DMSO.

### Single-Cell Ca^2+^ Measurements

Single-cell Ca^2+^ levels in MSNs were measured using the ratiometric Ca^2+^ indicator Fura-2 acetoxymethyl ester (Fura-2AM). Cells were grown on eight-well chamber slides and loaded with 2 μM Fura-2AM for 20 min at 37°C in a solution that contained 145 mM NaCl, 5 mM KCl, 0.75 mM Na_2_HPO_4_, 10 mM glucose, 10 mM HEPES (pH 7.4) and 1 mM MgCl_2_ Hank’s Balanced Salt Solution (HBSS) supplemented with 2 mM CaCl_2_ (high-Ca^2+^ medium) and then rinsed and left undisturbed for 15 min at 37°C to allow de-esterification. Measurements of intracellular Ca^2+^ levels were performed every 1 s at 37°C using Olympus Cell^∧^R imaging software, an IX81 microscope, a 10×/0.4 UPlanSApo objective (Olympus), and a Hamamatsu EM-CCD C9100-02 camera (Hamamatsu Photonics). Intracellular Ca^2+^ levels in individual neuronal cell bodies are expressed as the F340/F380 ratio after subtracting background fluorescence. This ratio represents the emission intensity at 510 nm that is obtained after excitation at 340 and 380 nm. The low-Ca^2+^ medium (Ca^2+^-free solution) contained 0.1 mM EGTA in standard buffer. To induce SOC channel activity, Corning BioCoat Poly-D-Lysine/Laminin Cellware 8-Well Culture Slides were applied. Data processing was performed using Olympus Cell^∧^R software, Microsoft Excel, and GraphPad Prism. For the single-cell Ca^2+^ measurements, the tetrahydrocarbazole compound was added for 5 min before starting the measurements at a 10 μM concentration in HBSS buffer.

SK-N-SH cells were grown on glass coverslips and loaded with 5 μM Fura-2AM (Invitrogen) in the presence of 0.025% Pluronic (Invitrogen) for 40 min at room temperature. Loaded cells were illuminated by alternating 340- and 380-nm excitation light at 2 Hz. The emission fluorescence intensity was measured at 510 nm using the InCyt Basic I/P dual wavelength fluorescence imaging system (Intracellular Imaging). Changes in cytosolic Ca^2+^ concentration were evaluated by calculating the ratio of emission fluorescence intensity at 340 and 380 nm excitation wavelengths (i.e., the 340/380 ratio).

### Electrophysiological Studies

Ion currents were recorded using the whole-cell patch-clamp technique (Hamill and Sakmann, 1981). The measurements were performed using an Axopatch 200B amplifier (Axon Instruments). The microelectrode resistance was 5–10 MΩ. The series resistance was uncompensated. Series resistance values were in the range of 10–25 MΩ and controlled during the entire experiment. The signal was enhanced, filtered by an internal two-pole Bessel filter (5 kHz section frequency), and digitized at 5 kHz using an AD converter plate (L-Card). During the recording of integral currents, the membrane potential was held at −40 mV. The membrane potential was periodically dropped every 5 s to −100 mV for 30 ms and then gradually increased to 100 mV at 1 mV/ms and then returned to −40 mV. Measurements were performed at 0.5-mV intervals. The recorded currents were normalized relative to cell capacitance (6–20 pF). The traces that were recorded before current activation were used as templates for leak subtraction. The pipette solution contained 125 mM CsCl, 10 mM EGTA-Cs, 30 mM HEPES-Cs, 4.5 mM CaCl_2_, 1.5 mM MgCl_2_, 4 mM Mg-adenosine triphosphate (ATP) and 0.4 mM Na-guanosine triphosphate (GTP), pH 7.3 (adjusted with CsOH). The extracellular solution contained 140 mM NMDG-Asp, 10 mM BaCl_2_, 30 mM HEPES-Cs and 0.01 mM nifedipine, pH 7.3 (adjusted with CsOH). Currents were evoked by the application of 1 μM thapsigargin (Tg, Sigma, catalog no. T9033) to the external solution. For the experiments that demonstrated the suppression of SOC currents, 1 μM gadolinium chloride (Sigma, catalog no. 439770) solution was used. All of the chemical compounds were provided by Sigma Aldrich.

### Statistical Analysis

The statistical analyses were performed using Prism 5.02 software (GraphPad). The data are expressed as the mean ± SEM from *n* MSNs or as average in at least three separate primary cultures. One-way analysis of variance (ANOVA) was used to analyze sets of single-cell Ca^2+^ measurement data. Tukey’s *post hoc* test was used to determine statistically significant differences among groups. Statistical comparisons of the results of the real-time PCR experiments with YAC128 and wildtype MSN cultures were performed using Student’s unpaired *t*-test. Values of *p* < 0.05 were considered statistically significant. To compare the amplitudes of SOC currents in different SK-N-SH cells, all of the currents were normalized to cell capacitance. Statistical comparisons were performed using one-way ANOVA with Bonferroni correction (normality and equal variances were confirmed by the Shapiro-Wilk and Levene tests, respectively). To compare the amplitudes of Gd^3+^-sensitive currents and levels of expression of the proteins, the Mann-Whitney-Wilcoxon test was used.

## Results

### mRNA Expression of Proteins That Are Involved in SOCE Is Unchanged in YAC128 MSN Cultures

**Figure 1:**
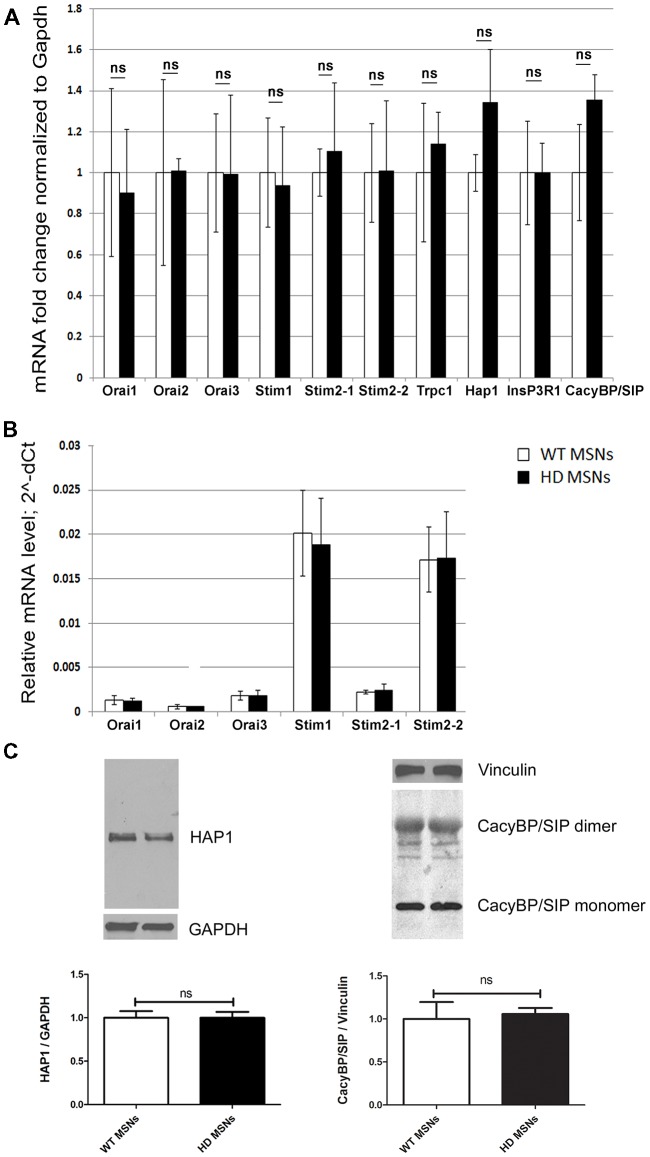
Analysis of mRNA that encode store-operated calcium entry (SOCE) proteins in day *in vitro* 21 (DIV21) medium spiny neuron (MSN) cultures from YAC128 and wildtype mice. **(A)** Gene expression analysis of mRNA that encode SOCE proteins and proteins that are involved in Ca^2+^ homeostasis in MSNs from wildtype (control) and YAC128 mice using real-time polymerase chain reaction (PCR). The gene expression results for all genes were normalized to *Gapdh*. Data from wildtype MSNs were normalized to 1. **(B)** Relative mRNA levels of genes that encode SOCE proteins, measured as 2^∧^-dCt. **(C)** Immunoblots of HTT-associated protein-1 (HAP1) normalized to GAPDH and CacyBP/SIP (both monomers and dimers) normalized to Vinculin in YAC128 and wildtype MSN cultures. On the bottom of each panel, the expression levels of HAP1 and CacyBP/SIP proteins are plotted. The results are expressed as mean ± SEM and represent data from three independent mRNA and protein preparations of three different MSN cultures. ns, not significant.

We previously found an approximately three-fold increase in HAP1 expression at the mRNA and protein levels in the striatum in 3-month-old YAC128 mice and an approximately two-fold increase in CacyBP/SIP (calcyclin-binding protein; Czeredys et al., 2013). To investigate whether HAP1 overexpression dysregulates SOCE in the present HD model, we switched to MSN cultures that were prepared from striata of DIV18 wildtype or hemizygous YAC128 mouse embryos and cultured for 3 weeks. Using real-time PCR, the mRNA levels of HAP1 were quantified in YAC128 MSNs and wildtype cultures. HAP1 mRNA levels increased in YAC128 MSNs, but this increase was not statistically significant (Figure 1A). We also evaluated levels of the mRNA expression of CacyBP/SIP, SOCE components, IP_3_R1 and TRPC1 and found no significant changes between the analyzed cultures (Figure 1A). Using Western blot, we detected no significant changes in HAP1 or CacyBP/SIP protein levels between YAC128 MSNs and wildtype cultures (Figure 1C). The difference in mRNA levels between brains from adult YAC128 mice (in which a large increase in some proteins was observed) and YAC128 MSN cultures (which exhibited no changes) was likely attributable to age. We assume that in YAC128 MSN cultures that are older than 3 weeks, mRNA upregulation would occur, but such cultures cannot be used for Ca^2+^ measurements. In 3 weeks cultures, we observed the simultaneous response of MSNs due to very high cell density and a large number of connections in the neural network and overgrown of glial cells in these cultures. Therefore, we overexpressed HAP1 protein in DIV14 YAC128 MSN cultures to mimic the conditions in the striatum in YAC128 mice *in vivo* and then verified its role in SOCE dysregulation.

**Figure 2:**
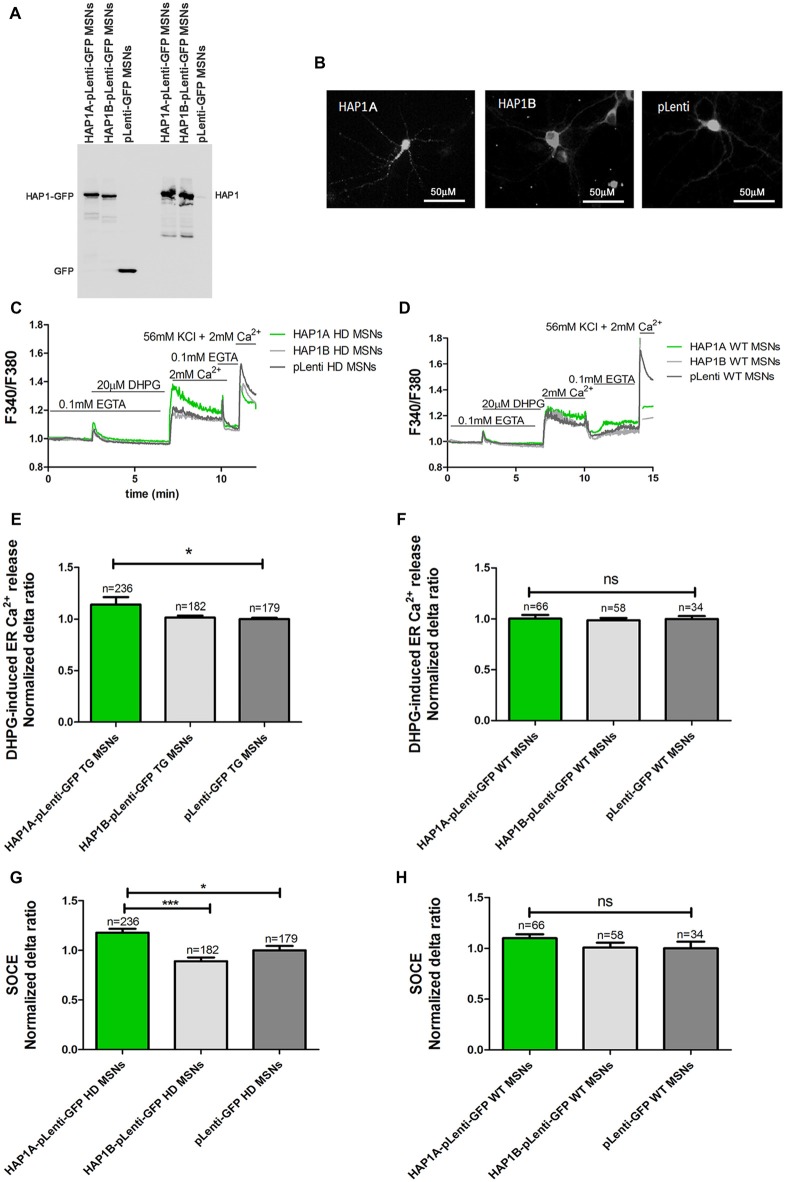
Parameters of Ca^2+^ homeostasis in YAC128 MSNs that overexpress HAP1A. **(A)** Immunoblots of HAP1 and green fluorescent protein (GFP) in YAC128 MSN cultures that overexpressed HAP1A-pLenti-GFP, HAP1B-pLenti-GFP and pLenti-GFP. **(B)** GFP fluorescence that indicates the overexpression of HAP1A-pLenti-GFP and HAP1B-pLenti-GFP and pLenti-GFP in YAC128 MSNs. **(C)** Protocol to induce SOCE in YAC128 MSNs that overexpressed HAP1 isoforms using the eight-well system. **(D)** Protocol to induce SOCE in wildtype MSNs that overexpressed HAP1 isoforms. Cultures of MSNs on DIV14 from YAC128 and wildtype mice (control) that overexpressed HAP1A-pLenti-GFP, HAP1B-pLenti-GFP, or pLenti-GFP using lentiviruses were loaded with the Ca^2+^ indicator Fura-2AM and incubated in Ca^2+^-free medium (0.1 mM EGTA). Ca^2+^ release from the endoplasmic reticulum (ER) was induced by 20 μM DHPG. SOCE was activated by the addition of 2 mM Ca^2+^ to the medium. KCl (56 nM) in 2 mM Ca^2+^ was applied to distinguish neurons from glial cells. **(E)** Ca^2+^ release from the ER in YAC128 MSNs that overexpressed HAP1 isoforms. **(F)** Ca^2+^ release from the ER in wildtype MSNs that overexpressed HAP1 isoforms. **(G)** Ca^2+^ influx via DHPG-induced SOCE in YAC128 MSNs that overexpressed HAP1 isoforms. **(H)** Ca^2+^ influx via DHPG-induced SOCE in wildtype MSNs that overexpressed HAP1 isoforms. The results are expressed as mean ± SEM. The number of cells is shown on the top of the bars. The results of at least three independent MSN culture preparations are shown. *p < 0.05, ***p < 0.001. ns, not significant.

The mouse genome has two HAP1 isoforms (HAP1A and HAP1B) that differ in their C-termini that are responsible for interactions with IP_3_R1 (Nasir et al., 1998; Tang et al., 2003). cDNA that encoded both isoforms were recloned from CMV6-ENTRY plasmids into pLenti-mGFP to efficiently overexpress HAP1A or HAP1B in both YAC128 MSN and wildtype cultures. The overexpression of HAP1A or HAP1B using plasmids in the lentivirus system is presented in Figure 2A. HAP1B protein had higher mobility in the gel compared with HAP1A, which was attributable to differences in the C-terminal part of the protein. The HAP1 isoforms had different intracellular localization after overexpression in YAC128 MSNs (Figure 2B). HAP1A had puncta localization in the cell body and dendrites in YAC128 MSNs compared with HAP1B, the expression of which was equally distributed in the cytoplasm of MSNs.

The relative level of mRNA encoding HAP1 in the brain structures from YAC128 and wildtype mice was analyzed using TaqMan low-density PCR arrays. We found that relative expression of mRNA encoding HAP1 was significantly higher in the striatum of YAC128 mice than in other analyzed brain structures such as motor cortex and cerebellum which are also known as brain structures involved in HD pathology (Supplementary Figure S1A). In wildtype mice, the relative expression of mRNA encoding HAP1 was significantly higher in the striatum and the hippocampus compared to other analyzed brain structures (Supplementary Figure S1B).

### HAP1A Regulates ER Ca^2+^ Release in MSNs From YAC128 Mice

HAP1A was shown to potentiate the effect of mutant HTT on IP_3_R1-mediated Ca^2+^ release in mouse MSNs (Tang et al., 2004). We verified the effect of HAP1A on the possible activation of IP_3_R1 in the present HD model by comparing Ca^2+^ signals in YAC128 and wildtype MSN cultures that overexpressed HAP1 isoforms. The Ca^2+^ measurements were performed on DIV14 in YAC128 and wildtype MSNs that overexpressed HAP1A-pLenti-GFP or HAP1B-pLenti-GFP and the control plasmid pLenti-GFP (Figures 2C,D, respectively). In all of the single-cell Ca^2+^ measurement experiments, we distinguished neurons from glial cells at the end of each experiment by applying 56 mM KCl. Neurons are characterized by a fast response to a high concentration of K^+^ ions, resulting in the opening of voltage-gated calcium channels (VGCCs) and the rapid influx of Ca^2+^ ions into the cytosol (Carmignoto et al., 1998). To examine the effect of overexpression of the HAP1A and HAP1B isoforms in YAC128 MSN cultures on IP_3_R1-mediated Ca^2+^ efflux from the ER, 20 μM DHPG (metabotropic glutamate receptor 1/5 (mGluR1/5) agonist that activates the PLC/IP_3_R signaling pathway was applied (Figure 2C). HAP1A protein overexpression significantly increased IP_3_R1-mediated Ca^2+^ efflux from the ER (*p* < 0.05), measured as the average delta ratio in YAC128 MSN cultures (1.13 ± 0.01) compared with the HAP1B isoform (1.0 ± 0.02) and control cells (1.0 ± 0.01; Figure 2E). We did not observe an effect of HAP1A overexpression on IP_3_R1-mediated Ca^2+^ efflux from the ER in wildtype MSNs that overexpressed this protein (Figure 2F).

**Figure 3:**
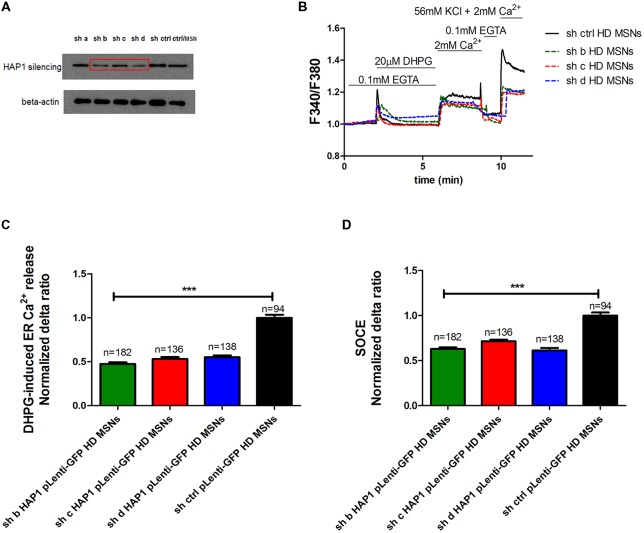
Parameters of Ca^2+^ homeostasis in YAC128 MSNs with silencing of HAP1A. **(A)** Immunoblots of HAP1 and beta-actin in YAC128 MSN cultures that overexpressed short-hairpin RNA (shRNA) against HAP1. shRNAs against HAP1 are named a, b, c, d, control scrambled shRNA, and control MSN cultures as indicated on the blot. **(B)** Protocol to induce SOCE in YAC128 MSNs with silencing of HAP1 using the eight-well system. Cultures of MSNs on DIV14 from YAC128 mice that overexpressed different shRNAs against HAP1 in pLenti-GFP were loaded with the Ca^2+^ indicator Fura-2AM and incubated in Ca^2+^-free medium (0.1 mM EGTA). Ca^2+^ release from the ER was induced by 20 μM DHPG. SOCE was activated by the addition of 2 mM Ca^2+^ to the medium. KCl (56 nM) in 2 mM Ca^2+^ was applied to distinguish neurons from glial cells. **(C)** Ca^2+^ release from the ER in YAC128 MSNs that overexpressed different shRNAs against HAP1. **(D)** Ca^2+^ influx via DHPG-induced SOCE in YAC128 MSNs that overexpressed different shRNAs against HAP1. The results are expressed as mean ± SEM. The number of cells is shown on the top of the bars. The results of three independent MSN culture preparations are shown. ***p < 0.001.

To confirm that HAP1 protein activates IP_3_R1-mediated Ca^2+^ efflux from the ER in the presence of mutant HTT, we used gene silencing technology. The effects of four different shRNAs against HAP1 were checked by Western blot and compared with control shRNA (Figure 3A). We found that three shRNAs (indicated by b, c and d in Figure 3A) most effectively decreased HAP1 protein levels by approximately 0.53-fold, 0.4-fold, and 0.58-fold, respectively. shRNA indicated by a reduced HAP1 levels only 0.9-fold.

**Figure 4:**
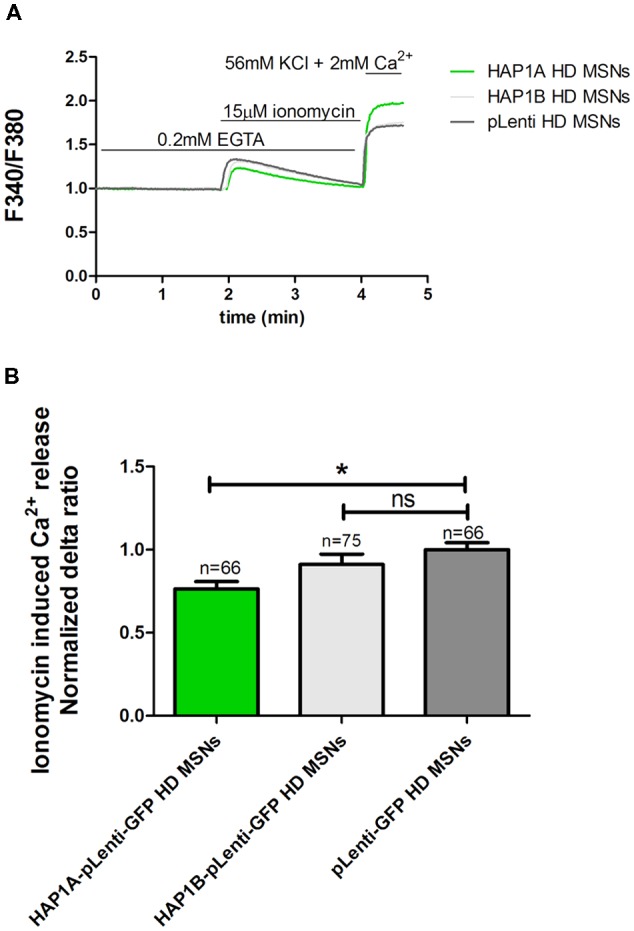
Effect of HAP1A on ER Ca^2+^ content in YAC128 MSNs. Cultures of MSNs on DIV14 from YAC128 mice that overexpressed HAP1A-pLenti-GFP, HAP1B-pLenti-GFP, or pLenti-GFP using lentiviruses that were loaded with the Ca^2+^ indicator Fura-2AM. **(A)** The figure shows the protocol to induce ionomycin-induced Ca^2+^ release from the ER in YAC128 MSNs that overexpressed HAP1 isoforms using the eight-well system. YAC128 MSNs that overexpressed HAP1A isoforms were incubated in Ca^2^-free medium (0.1 mM EGTA), followed by 15 μM ionomycin application to induce the release of Ca^2+^. KCl (56 nM) in 2 mM Ca^2+^ was applied to distinguish neurons from glial cells. **(B)** Ionomycin-induced ER Ca^2+^ release in YAC128 MSNs that overexpressed HAP1 isoforms. The results are expressed as mean ± SEM. The number of cells is shown on the top of the bars. *p < 0.05. ns, not significant. The results were obtained from three independent MSN culture preparations.

To examine the effect of HAP1 silencing in YAC128 MSN cultures on IP_3_R1-mediated Ca^2+^ efflux from the ER, 20 μM DHPG was applied as shown in Figure 3B. The silencing of HAP1 by three separate shRNAs (b, c and d) in YAC128 MSN cultures significantly decreased (*p* < 0.001) IP_3_R1-mediated Ca^2+^ efflux from the ER, measured as the normalized delta ratio. The delta ratios were 0.47 ± 0.01, 0.53 ± 0.02 and 0.55 ± 0.02, respectively, for these three shRNAs against HAP1 compared with cells that were transduced by scrambled shRNA (1.0 ± 0.03; Figure 3C). Based on these experiments, we reasoned that the activation of IP_3_R1 under physiological conditions may lead to greater ER Ca^2+^ leakage and lower steady-state ER Ca^2+^ levels in YAC128 MSNs that overexpress HAP1A. To examine the effect of HAP1A overexpression on ER Ca^2+^ content in striatal cultures, we measured the ionomycin-sensitive ER Ca^2+^ pool in YAC128 MSN cultures that were transduced by HAP1A lentiviruses compared with HAP1B and pLenti-GFP as a control (Figure 4A). When we applied 15 μM ionomycin (Sigma, catalog no. I0634) to YAC128 MSNs that overexpressed HAP1A, significantly lower ER Ca^2+^ content was observed (*p* < 0.05) compared with YAC128 MSNs that were transduced by viruses with HAP1B or control plasmid (Figure 4B). The delta ratio of ionomycin-induced Ca^2+^ release was 0.76 ± 0.04 in YAC128 MSNs that overexpressed HAP1A, 0.91 ± 0.06 in YAC128 MSNs that overexpressed HAP1B, and 1 ± 0.04 in MSNs that overexpressed pLenti-GFP as a control. These data support the hypothesis that the supranormal steady-state activity of IP_3_R1 is a major cause of greater ER Ca^2+^ leakage and lower ER Ca^2+^ levels in YAC128 MSNs.

### Overexpression of HAP1A Increases DHPG-Induced SOCE in MSNs From YAC128 Mice

One of the pathological hallmarks of HD is an elevation of SOCE. We analyzed the effect of higher levels of HAP1 on the possible dysfunction of SOCE. Ca^2+^ signals in YAC128 and wildtype MSN cultures that overexpressed HAP1 isoforms were examined. Ca^2+^ measurements were performed on DIV14 MSNs that overexpressed HAP1A-pLenti-GFP, HAP1B-pLenti-GFP, or pLenti-GFP (control plasmid). We first measured Ca^2+^ ion entry into the cytosol in YAC128 (Figure 2C) and wildtype (Figure 2D) MSNs that overexpressed HAP1 isoforms. IP_3_R1-mediated Ca^2+^ efflux from the ER was induced by 20 μM DHPG. This treatment was followed by the addition of 2 mM Ca^2+^ to the extracellular medium, which resulted in Ca^2+^ influx via SOCE (Figures 2C,D). We detected a significant increase in DHPG-induced SOCE in YAC128 MSNs that overexpressed HAP1A-pLenti-GFP compared with neurons that overexpressed pLenti-GFP (*p* < 0.05) and HAP1B-pLenti-GFP (*p* < 0.001; Figure 2G), but this effect was not observed in wildtype MSN cultures that overexpressed HAP1A protein (Figure 2H). The average normalized delta ratio of SOCE was 1.18 ± 0.04 for YAC128 MSNs that overexpressed HAP1A-pLenti-GFP, 0.89 ± 0.03 for YAC128 MSNs that overexpressed HAP1B-pLenti-GFP, and 1.00 ± 0.04 for YAC128 MSNs that overexpressed pLenti-GFP as a control (Figure 2G).

**Figure 5:**
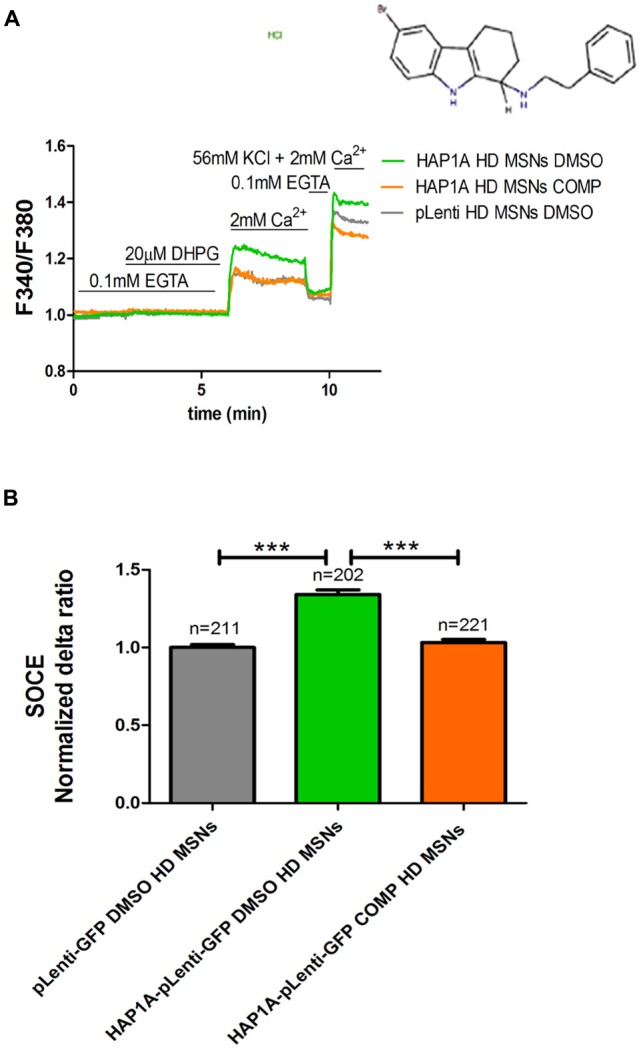
Effect of tetrahydrocarbazole on Ca^2+^ signals in MSNs that overexpress HAP1A. MSN cultures on DIV14 from YAC128 mice that overexpressed HAP1A-pLenti-GFP or pLenti-GFP as a control were loaded with the Ca^2+^ indicator Fura-2AM and incubated in Ca^2+^-free medium. The MSN cultures were incubated for 5 min with tetrahydrocarbazole or dimethylsulfoxide (DMSO; control) in Ca^2+^-free medium. **(A)** The figure shows the protocol to measure DHPG-induced SOCE using the eight-well system. The structural formula of 6-bromo-*N*-(2-phenylethyl)-2,3,4,9-tetrahydro-1*H*-carbazol-1-amine hydrochloride is shown. The effect of tetrahydrocarbazole on DHPG-induced SOCE in YAC128 MSNs that overexpressed HAP1A-pLenti-GFP was measured followed by the re-addition of 2 mM Ca^2+^ to the medium compared with YAC128 MSNs that overexpressed HAP1A-pLenti-GFP or pLenti-GFP and were treated with 0.02% DMSO as a control. KCl (56 nM) in 2 mM Ca^2+^ was applied to distinguish neurons from glial cells at the end of the protocol. **(B)** Ca^2+^ influx via DHPG-induced SOCE in YAC128 MSNs overexpressed HAP1A-pLenti-GFP isoform and were treated with tetrahydrocarbazole compared with YAC128 MSNs that overexpressed HAP1A-pLenti-GFP or pLenti-GFP that were treated with 0.02% DMSO. The results are expressed as mean ± SEM. The number of cells is shown on the top of the bars. ***p < 0.001. The results were obtained from three independent MSN culture preparations.

We previously showed that tetrahydrocarbazoles stabilize SOCE in YAC128 MSN cultures (Czeredys et al., 2017). In the present study, we investigated whether the most effective tetrahydrocarbazole in decreasing SOCE (6-bromo-*N*-[2-phenylethyl]-2,3,4,9-tetrahydro-1*H*-carbazol-1-amine hydrochloride [C_20_H_22_BrClN_2_]) is able to stabilize disturbances in SOCE upon HAP1A overexpression. YAC128 MSN cultures that overexpressed HAP1A were treated with 10 μM C_20_H_22_BrClN_2_ for 5 min before performing Ca^2+^ measurements according to the protocol that is shown in Figure 5A. MSN cultures that overexpressed HAP1A-pLenti-GFP or pLenti-GFP and were treated with 0.02% DMSO were used as a control. We found that C_20_H_22_BrClN_2_ significantly normalized the average delta ratio of SOC channel activity (*p* < 0.001) up to 1.05 ± 0.01 in YAC128 MSN cultures that overexpressed HAP1A (Figure 5B) compared with cells that overexpressed HAP1A and were treated with 0.02% DMSO (1.4 ± 0.03). After treatment with C_20_H_22_BrClN_2_, the level of SOC channel activity in YAC128 MSN cultures that overexpressed HAP1A was indistinguishable from MSN cultures that overexpressed pLenti-GFP and were treated with 0.02% DMSO (1.0 ± 0.02; Figure 5B).

We used gene silencing technology to examine whether the lower level of HAP1 protein in YAC128 MSN cultures affects SOCE. Three individual viruses with shRNAs against HAP1 in pLenti-GFP (b, c, d) and scrambled shRNA in pLenti-GFP as a control were transduced in YAC128 cultures, and SOC channel activity was measured in DIV14 MSNs as shown in Figure 3B. The silencing of HAP1 using these three shRNAs against HAP1 in YAC128 MSN cultures significantly decreased the average delta ratio of SOCE (*p* < 0.001). We observed a decrease in SOCE to 0.63 ± 0.01 for HAP1 shRNA b, 0.71 ± 0.01 for HAP1 shRNA c, and 0.62 ± 0.02 for HAP1 shRNA d compared with control cells that were transduced by scrambled shRNA (1.0 ± 0.03; Figure 3D).

### Inhibition of IP_3_R1 Normalizes Elevation of SOCE in MSNs From YAC128 Mice

**Figure 6:**
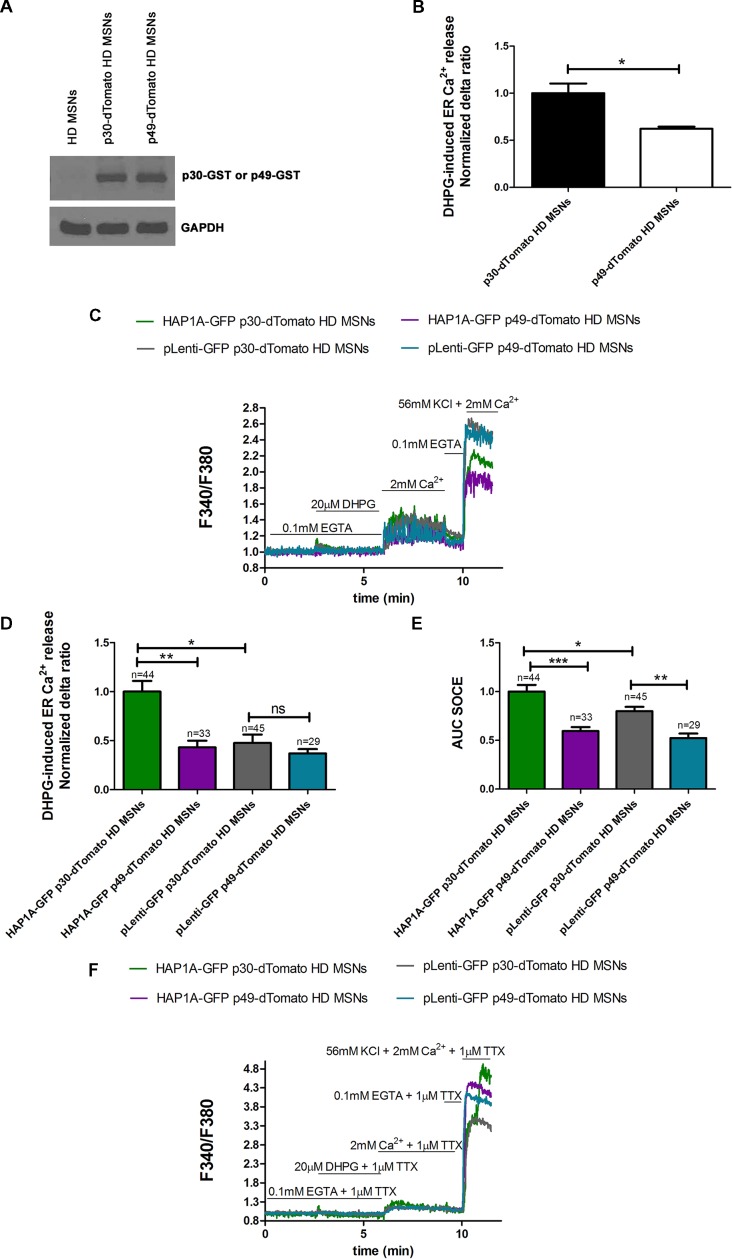
Effect of inositol-(1,4,5)triphosphate (IP_3_) sponge inhibitor on Ca^2+^ signaling in MSNs that overexpress HAP1A. **(A)** Immunoblots of YAC128 MSN cultures that overexpressed the glutathione *S*-transferase (GST)-tagged IP_3_R1 ligand binding region (226–604 amino acid region) with a point mutation (R441Q: m49) cloned into the pUltra-Chili vector, indicated as p49-GST, and the negative control with a point mutation (K508A: m30), indicated as p30-GST cloned into the pUltra-Chili vector or control (non-transduced) MSNs. MSN cultures on DIV14 from YAC128 mice that overexpressed p49-dTomato and p30-dTomato as an IP_3_ sponge and control respectively were loaded with the Ca^2+^ indicator Fura-2AM and incubated in Ca^2+^-free medium. Ca^2+^ release from the ER was induced by 20 μM DHPG. **(B)** DHPG-induced Ca^2+^ release from the ER in YAC128 MSNs that overexpressed p49-dTomato or p30-dTomato as a control for the IP_3_ sponge inhibitor. **(C)** Protocol to measure DHPG-induced ER Ca^2+^ release and DHPG-induced SOCE using the eight-well system. MSN cultures on DIV14 from YAC128 mice that overexpressed HAP1A-pLenti-GFP or pLenti-GFP and p49-dTomato or p30-dTomato were loaded with Fura-2AM and incubated in Ca^2^-free medium. Ca^2+^ release from the ER was induced by 20 μM DHPG. SOCE was activated by the addition of 2 mM Ca^2+^ to the medium. KCl (56 nM) in 2 mM Ca^2+^ was applied to distinguish neurons from glial cells. **(D)** Effect of p49-dTomato or p30-dTomato on DHPG-induced ER Ca^2+^ release in YAC128 MSNs that overexpressed HAP1A-pLenti-GFP or pLenti-GFP. **(E)** Effect of p49-dTomato or p30-dTomato on DHPG-induced SOCE in YAC128 MSNs that overexpressed HAP1A-pLenti-GFP or pLenti-GFP. **(F)** To avoid the firing-effect in MSNs, 1 μM tetrodotoxin (TTX) was added during the Ca^2+^ measurement protocol. In the presence of TTX, DHPG-induced ER Ca^2+^ release and DHPG-induced SOCE were measured using the eight-well system. MSN cultures on DIV14 from YAC128 mice that overexpressed HAP1A-pLenti-GFP or pLenti-GFP and p49-dTomato or p30-dTomato were loaded with Fura-2AM and incubated in Ca^2+^-free medium. Ca^2+^ release from the ER was induced by 20 μM DHPG. SOCE was activated by the addition of 2 mM Ca^2+^ to the medium. KCl (56 nM) in 2 mM Ca^2+^ was applied to distinguish neurons from glial cells. The results are expressed as mean ± SEM. The number of cells is shown on the top of the bars. Normalized delta ratio was analyzed as average from three experiments **(B,D)**. *p < 0.05, **p < 0.01, ***p < 0.001. ns, not significant. The results were obtained from three independent MSN culture preparations.

To confirm that the elevation of SOCE in MSN cultures that overexpressed HAP1A resulted from greater IP_3_R1 activation by HAP1A, we used the IP_3_ sponge inhibitor p49, which was found to specifically decrease the activity of IP_3_R1 in HEK293 (Uchiyama et al., 2002) cells and starfish oocytes (Iwasaki et al., 2002). p49 exhibits ~1,000-times higher IP_3_ binding activity than endogenous IP_3_R1 compared with the control variant p30 that has low affinity for IP_3_ (Iwasaki et al., 2002; Uchiyama et al., 2002). We confirmed the appropriate cloning and protein expression of the IP_3_ sponge inhibitor and control IP_3_R1 fragment using Western blot. With the application of anti-GST antibody, we detected the overexpression of p49-GST and p30-GST fusion proteins in pUltra-Chili vectors in YAC128 MSNs compared with non-transduced cells (Figure 6A). During the Ca^2+^ measurements, YAC128 MSNs that overexpressed IP_3_ sponge or control were identified by dTomato fluorescence expression. In the control experiments, we confirmed that the IP_3_ sponge inhibitor specifically reduced the IP_3_R1-mediated release of Ca^2+^ from the ER in YAC128 MSN cultures, since IP_3_R1 is a dominant IP_3_R isoform in the striatum of HD mice (Supplementary Figure S2A). We found that relative expression of mRNA encoding IP3R1 was lower in the striatum comparing to the cerebellum of YAC128 mice (Supplementary Figure S2B). In YAC128 MSNs that overexpressed p49-dTomato, DHPG-induced IP_3_R1 activity (measured as the normalized delta ratio) significantly decreased (*p* < 0.05) to 0.62 ± 0.02 compared with control cells that overexpressed p30-dTomato (1.0 ± 0.1; Figure 6B).

To confirm that HAP1A activates SOCE by increasing IP_3_R1 activity in the present HD model, YAC128 MSN cultures that overexpressed HAP1A-pLenti-GFP or pLenti-GFP as a control and p49-dTomato or p30-dTomato corresponding to GST-tagged IP3R1 sponge or a control fragment were used for the Ca^2+^ measurements that were performed according to the protocol that is shown in Figure 6C. MSNs that expressed both GFP and dTomato tags were analyzed. The overexpression of p49-dTomato in YAC128 MSNs that overexpressed HAP1A-pLenti-GFP significantly stabilized (*p* < 0.01) DHPG-induced ER Ca^2+^ release, measured as a normalized delta ratio of 0.43 ± 0.06 compared with cells that overexpressed both HAP1A-pLenti-GFP and p30-dTomato (1.0 ± 0.11; Figure 6D). The delta ratio of Ca^2+^ release from the ER after inhibition by IP_3_ sponge in YAC128 MSNs that overexpressed HAP1A-pLenti-GFP was similar to YAC128 MSNs that overexpressed both pLeni-GFP and p30-dTomato (0.48 ± 0.08). We analyzed this dataset by measuring the area under the curve (AUC). DHPG-induced SOCE was significantly decreased (*p* < 0.001) in YAC128 MSNs that overexpressed both HAP1A-pLenti-GFP and p49-dTomato compared with cells that were transduced with both HAP1A-pLenti-GFP and p30-dTomato (Figure 6E). The average AUCs for these cells were 0.59 ± 0.04 and 1.0 ± 0.06, respectively. Moreover, the average AUC of SOCE after inhibition by the IP_3_ sponge inhibitor in YAC128 MSNs that overexpressed HAP1A was even lower than in YAC128 MSNs that overexpressed both pLeni-GFP and p30-dTomato (0.8 ± 0.04). HAP1A increased both DHPG-induced IP_3_R1 activity and SOCE (both *p* < 0.05) in cells that overexpressed HAP1A-pLenti-GFP and p30-dTomato *vs*. pLenti-GFP and p30-dTomato (Figures 6D,E). DHPG-induced SOCE in YAC128 MSNs that overexpressed HAP1A-pLenti-GFP and p30-dTomato was 1.0 ± 0.06 compared with pLenti-GFP and p30-dTomato (0.8 ± 0.04; Figure 6E). DHPG-induced ER Ca^2+^ release in YAC128 MSNs that overexpressed HAP1A-pLenti-GFP and p30-dTomato was 1.0 ± 0.11 compared with pLenti-GFP and p30-dTomato (0.48 ± 0.08; Figure 6D). The effect of oscillation in Ca^2+^ signal observed in MSNs overexpressed p49-dTomato (Figure 6C) could be eliminated by application of voltage-gated sodium channels blocker 1 μM tetrodotoxin (TTX; Figure 6F).

### Expression of HAP1A in SK-N-SH HTT138Q Results in the Appearance of Constitutively Active SOC Channels

**Figure 7:**
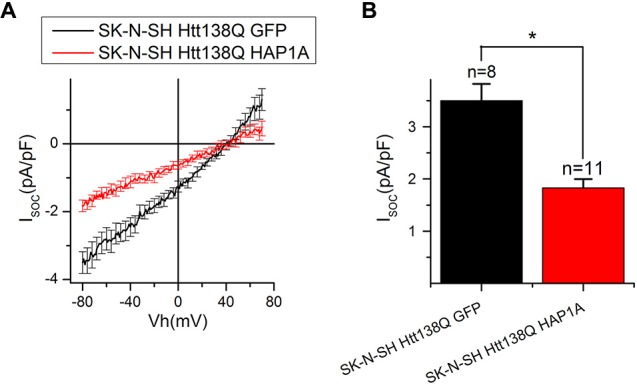
Reduction of thapsigargin-induced Ca^2+^ entry in SK-N-SH Htt138Q HAP1A. **(A)** Average current/voltage relationships (I/V curves) for currents that were evoked by passive Ca^2+^ store depletion with 1 μM thapsigargin in SK-N-SH Htt138Q cotransfected with GFP (black curve) or HAP1A (red curve). The number of experiments is shown in **(B)** above the bars. **(B)** Average amplitudes of thapsigargin-induced currents in SK-N-SH Htt138Q cotransfected with GFP (black bar) or HAP1A (red bar). Current amplitudes were determined at a test potential of −80 mV and plotted as mean ± SEM. *p < 0.05, significant difference in current amplitude.

To further investigate the effect of HAP1A expression on SOCE, we performed patch-clamp experiments by employing the previously described HD model based on the exogenous expression of full-length mutant HTT138Q in human neuroblastoma cells (SK-N-SH; Wu et al., 2011). SOCE was induced by the application of Tg, which blocks the sarcoendoplasmic reticulum calcium transport adenosine triphosphatase (SERCA) pump and causes passive Ca^2+^ store depletion. Electrophysiological recordings in whole-cell mode showed that the amplitude of Tg-induced currents in SK-N-SH HTT138Q cells that were co-transfected with GFP (SK-N-SH HTT138Q GFP) was 3.50 ± 0.32 pA/pF at −80 mV (Figures 7A,B). These data correlate well with previous results of SOCE measurements in SK-N-SH HTT138Q cells (Wu et al., 2011). The expression of HAP1A in SK-N-SH HTT138Q cells (SK-N-SH HTT138Q HAP1A) decreased the amplitude of Tg-induced SOCE to 1.83 ± 0.17 pA/pF (Figures 7A,B).

We then investigated whether the observed decrease in SOCE was a result of the analysis of patch-clamp recordings. To distinguish the currents through SOC channels, we subtracted currents that were recorded before Tg application. Therefore, if some of the SOC channels in SK-N-SH HTT138Q HAP1A were active before Tg treatment, then they would be subtracted during the analysis. The following approach was used, which is frequently applied in electrophysiological studies. The currents of interest were evoked until a maximum was reached, and then the currents were blocked with specific inhibitors. To obtain the I/V curve of inhibitor-sensitive currents, the final currents after full blockade were subtracted from the maximal currents (Kojima et al., 2012; Wei et al., 2017). To inhibit Tg-induced currents, we used Gd^3+^, which has been repeatedly shown to effectively block SOC channels in different cells (Bird et al., 2008; Wei et al., 2017).

Our hypothesis was that HAP1A expression strongly destabilizes IP_3_R1 in SK-N-SH HTT138Q and causes partial store depletion in response to a basal concentration of IP_3_ in cells, consequently leading to the activation of a portion of SOC channels. The application of Tg then induces complete store depletion and activation of the remaining SOC channels.

**Figure 8:**
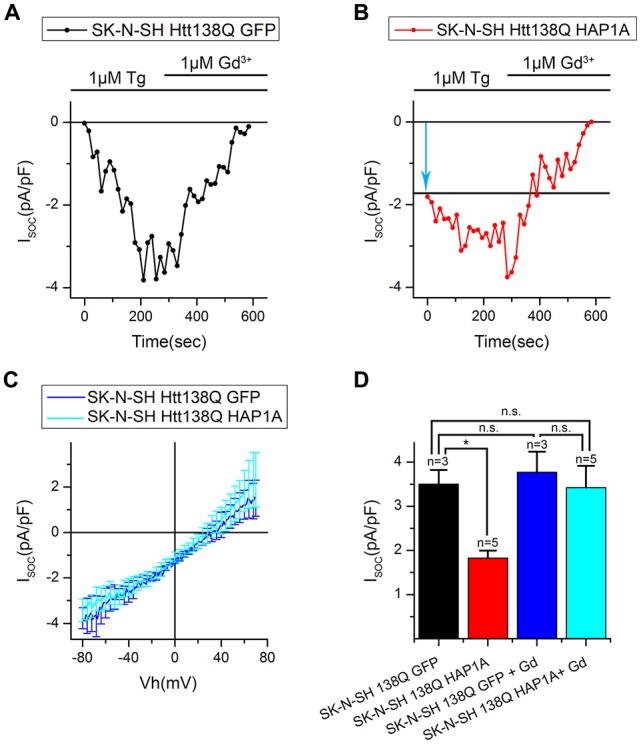
Constitutive activity of SOC channels in SK-N-SH Htt138Q HAP1A. **(A,B)** Amplitudes of thapsigargin-induced store-operated calcium currents in SK-N-SH Htt138Q cotransfected with GFP (**A**, black curve) or HAP1A (**B**, red curve) at a test potential of −80 mV plotted as a function of time. The times of thapsigargin and gadolinium application are indicated above the graphs. The level of the constitutive activity of SOC channels is indicated by the blue arrow in **(B)**. **(C)** Average current/voltage relationships (I/V curves) for gadolinium-sensitive currents in SK-N-SH Htt138Q cotransfected with GFP (dark blue curve) or HAP1A (light blue curve). The number of experiments is shown in **(D)** above the bars. **(D)** Average amplitudes of thapsigargin-induced currents in SK-N-SH Htt138Q cotransfected with GFP (black bar) or HAP1A (red bar) and gadolinium-sensitive currents in SK-N-SH Htt138Q cotransfected with GFP (dark blue bar) or HAP1A (light blue bar). Current amplitudes were determined at a test potential of −80 mV and plotted as mean ± SEM. *p < 0.05, significant difference in current amplitude. ns, not significant.

The results of the electrophysiological recordings showed that the amplitudes of Gd^3+^-sensitive currents in SK-N-SH HTT138Q GFP and SK-N-SH HTT138Q HAP1A were 3.77 ± 0.46 pA/pF and 3.42 ± 0.49 pA/pF, respectively, at −80 mV. Data from the same experiments showed that the amplitudes of Tg-induced currents were 3.50 ± 0.32 pA/pF and 1.82 ± 0.16 pA/pF, respectively, at −80 mV (Figures 8A–D). We found no significant differences in Gd^3+^-sensitive currents in SK-N-SH HTT138Q GFP and SK-N-SH HTT138Q HAP1A. Thus, the decrease in Tg-induced currents in SK-N-SH HTT138Q HAP1A compared with SK-N-SH HTT138Q GFP could be explained by the activation of a portion of SOC channels before Tg treatment. We concluded that the expression of HAP1A in SK-N-SH HTT138Q cells results in the appearance of constitutively active SOC channels without any additional incentives.

**Figure 9:**
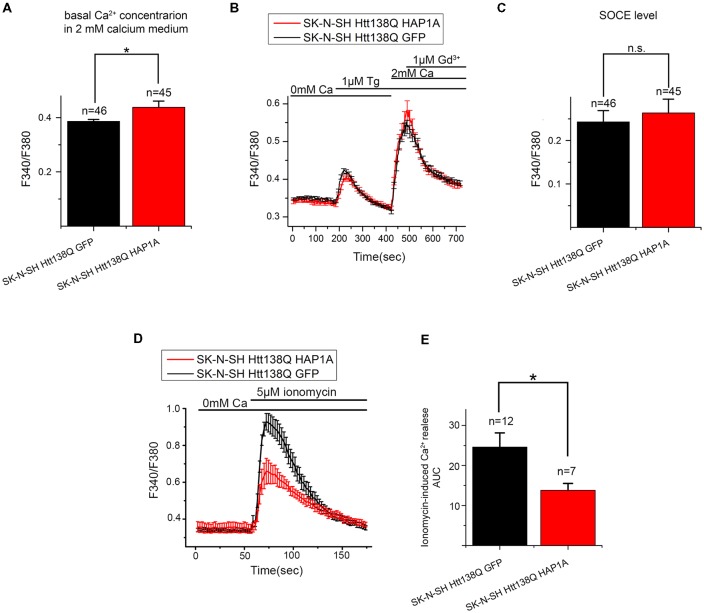
Level of SOCE in SK-N-SH Htt138Q HAP1A. **(A)** Average basal Ca^2+^ levels in the medium that contained 2 mM CaCl_2_ in SK-N-SH Htt138Q cotransfected with GFP (black bar) or HAP1A (red bar), plotted as mean ± SEM. *p < 0.05, significant difference in basal calcium level. The number of experiments is shown above the bars. **(B)** Responses of cytosolic calcium levels to 1 μM thapsigargin application in SK-N-SH Htt138Q cotransfected with GFP (black curve) or HAP1A (red curve). The number of experiments is shown in **(C)** above the bars. **(C)** Average amplitudes of thapsigargin-induced SOCE levels in SK-N-SH Htt138Q cotransfected with GFP (black bar) or HAP1A (red bar), plotted as mean ± SEM. *p < 0.05, significant difference in current amplitude. **(D)** Responses of cytosolic calcium levels to the application of 5 μM ionomycin in SK-N-SH Htt138Q cotransfected with GFP (black curve) or HAP1A (red curve). The number of experiments is shown in **(E)** above the bars. **(E)** Average amplitudes of ionomycin-induced calcium release levels in SK-N-SH Htt138Q cotransfected with GFP (black bar) or HAP1A (red bar), plotted as mean ± SEM. *p < 0.05, significant difference in current amplitude. ns, notsignificant.

To confirm this possibility, we performed additional experiments using the fluorescent calcium dye Fura-2AM. Basal Ca^2+^ levels that were measured in 2 mM Ca^2+^ external solution was higher in SK-N-SH HTT138Q HAP1A than in SK-N-SH HTT138Q GFP (Figure 9A). This supported our hypothesis that HAP1A may cause partial Ca^2+^ store depletion and the consequent activation of a portion of SOC channels in SK-N-SH HTT138Q. Nevertheless, no significant differences in Tg-induced Ca^2+^ store depletion were found between SK-N-SH HTT138Q GFP and SK-N-SH HTT138Q HAP1A (Figure 9B). The Fura-2AM Ca^2+^ imaging data indicated that the amplitudes of Tg-induced SOCE (Figures 9B,C) were similar in SK-N-SH HTT138Q GFP and SK-N-SH HTT138Q HAP1A. This result confirms that full entry through SOC channels in SK-N-SH HTT138Q GFP and SK-N-SH HTT138Q HAP1A after complete Ca^2+^ store depletion is equal and also equally sensitive to treatment with Gd^3+^ (Figure 9B).

We also used calcium imaging experiments to estimate the total Ca^2+^ store content by inducing Ca^2+^ efflux from stores to the cytoplasm with ionomycin. The experiments showed that ionomycin-induced Ca^2+^ release was significantly higher in SK-N-SH HTT138Q GFP comparing to SK-N-SH HTT138Q HAP1A (Figures 9D,E). Thus, we confirmed that ER Ca^2+^ level is lower in cells overexpressing HAP1A.

### Expression of HAP1A in SK-N-SH HTT138Q Cells Affects STIM2 Expression Level

HAP1 has been shown to be involved in the regulation of gene expression (Wu and Zhou, 2009), suggesting that the expression of HAP1A in SK-N-SH HTT138Q results in changes in the proteome. We observed alterations of SOCE in SK-N-SH HTT138Q HAP1A; therefore, we investigated the expression levels of proteins that are responsible for SOCE in these cells.

**Figure 10:**
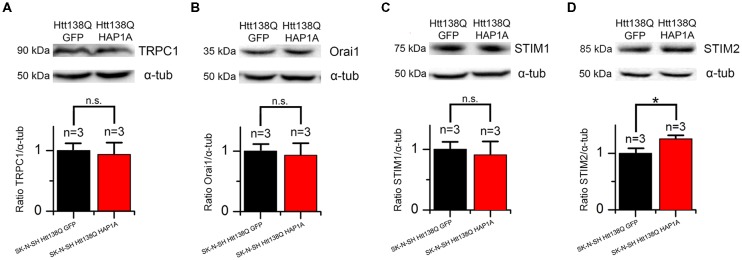
Expression levels of proteins that are involved in SOCE in SK-N-SH Htt138Q HAP1A. **(A–D)** Expression levels of transient receptor potential channel type 1 (TRPC1) **(A)**, Orai1 **(B)**, STIM1 **(C)** and STIM2 **(D)** proteins (normalized to α-tubulin) in SK-N-SH Htt138Q cotransfected with GFP (black bars) or HAP1A (red bars). On the top of each panel, the representative Western blots are shown. On the bottom of each panel, the expression levels of proteins are plotted as mean ± SEM. *p < 0.05, significant difference in expression level. ns, not significant. The number of tested lysates is shown above the bars.

We previously demonstrated that the main protein that maintains SOCE in these cells is the channel-forming protein TRPC1 (Wu et al., 2011). To reveal possible specific features of TRPC1 protein expression in SK-N-SH HTT138Q cells that are enriched in HAP1A, total lysates of cells that expressed HTT138Q and GFP or HTT138Q and HAP1A were analyzed by Western blot with antibodies to TRPC1. No differences in the levels of TRPC1 protein were found between cells (Figure 10A).

Orai1 protein forms highly selective CRAC channels with a low conductivity that contribute to SOCE in different cells, including neurons (Hoth and Penner, 1992; Parekh and Putney, 2005; Vig and Kinet, 2007; Prakriya and Lewis, 2015; Putney, 2017). No changes in the levels of this protein were found in cells that expressed HTT138Q and GFP or HTT138Q and HAP1A (Figure 10B).

The Ca^2+^ sensor STIM1 in the ER is one of the main players in SOCE machinery (Dziadek and Johnstone, 2007; Prakriya and Lewis, 2015; Putney, 2017). STIM1 protein was previously shown to be required for SOCE activation in SK-N-SH HTT138Q (Vigont et al., 2014). The expression levels of STIM1 were found to be the same in SK-N-SH cells that expressed HTT138Q and GFP or HTT138Q and HAP1A (Figure 10C).

STIM2 is a calcium sensor in the ER that regulates resting Ca^2+^ levels in the ER and Ca^2+^ leakage. Because of the lower level of intracellular Ca^2+^, STIM2 is sufficient to trigger STIM2-ORAI1 complex formation (Gruszczynska-Biegala et al., 2011; Gruszczynska-Biegala and Kuznicki, 2013). In the present cellular model of HD, we found that HAP1A overexpression increased STIM2 protein levels. The expression levels of STIM2 significantly increased (*p* < 0.05) in SK-N-SH cells that expressed HTT138Q and HAP1A compared with SK-N-SH cells that were transfected with HTT138Q and GFP (Figure 10D).

Thus, the decrease in Ca^2+^ entry through SOC channels as a result of the constitutive activity of SOC channels in SK-N-SH HTT138Q HAP1A cells may be related to changes in the expression levels of STIM2 protein.

## Discussion

### HAP1A Modulates IP_3_R1 Activity and Causes the Depletion of ER Ca^2+^

We previously found that ER Ca^2+^ release is elevated in YAC128 MSNs (Czeredys et al., 2017). In the present study, we found that overexpression of the isoform HAP1A but not HAP1B was important in modulating IP_3_R1 activity in the presence of mutHTT in YAC128 MSN cultures. HAP1A overexpression caused an increase in the activation of IP_3_R1 and consequently potentiated the depletion of ER Ca^2+^stores whereas the silencing of HAP1 decreased ER Ca^2+^ release in YAC128 MSNs. DHPG-induced ER Ca^2+^ release was elevated upon HAP1A overexpression in YAC128 MSN cultures but not in wildtype MSNs. These results confirmed previous findings, in which HAP1A interacted with the C-terminus of IP_3_R1 (Tang et al., 2003) and facilitated the potentiation of IP_3_R1-mediated Ca^2+^ release by mutHTT in mouse MSNs (Tang et al., 2004). The ternary complex that consists of IP_3_R1-HAP1A-Htt is formed *in vitro* and *in vivo* (Tang et al., 2003). Moreover, IP_3_R1 was shown to form a macro signal complex that contains, among other proteins, HAP1A protein and to function as a center for signaling cascades (Mikoshiba, 2007). Therefore, we can assume that HAP1A is a link between mutHTT and IP_3_R1 and can enhance the toxic effects of mutHTT on IP_3_R1 in YAC128 MSN cultures. We found that the activation of IP_3_R1 by DHPG enhanced Ca^2+^ release from the ER. We also found lower steady-state ER Ca^2+^ levels in YAC128 MSNs that overexpressed HAP1A, indicated by measurements of the ionomycin-sensitive ER Ca^2+^ pool in YAC128 MSN cultures that overexpressed HAP1A.

### HAP1A Affects SOCE in YAC128 MSNs

Disturbances in SOCE were observed by several groups that used different models of HD (Wu et al., 2011, 2016; Vigont et al., 2015; Czeredys et al., 2017). In the present study, we found that the HAP1A isoform acted as a regulator of SOC channels activity in the presence of mutHTT in YAC128 MSN cultures, in which HAP1A overexpression increased IP_3_R1 activation and consequently depleted ER Ca^2+^ stores. HAP1 protein overexpression was previously found in the striatum in adult YAC128 mice (Czeredys et al., 2013). HAP1 interacts directly with mutHTT, and its role in IP_3_R1 activation was observed in MSNs from HAP1^−/–^ transgenic mice (Tang et al., 2003, 2004). We were interested in whether HAP1 overexpression modulates SOC channels activity. Two HAP1 protein isoforms have been identified in rodents. These two isoforms, HAP1A and HAP1B, differ in their carboxy termini, which have the possibility of binding Htt (Li et al., 1995; Nasir et al., 1998). Importantly, the HAP1A isoform shares homology with human HAP1 (Li et al., 1998).

DHPG is a compound that may activate IP_3_R1 and induce SOCE (Vanderklish and Edelman, 2002; Ng et al., 2011; González-Sánchez et al., 2017). In the present study, we found that only the HAP1A isoform modulated DHPG-induced SOCE in YAC128 MSN cultures. This is consistent with previous studies that showed that only HAP1A interacts with IP_3_R1 and increases ER Ca^2+^ release (Tang et al., 2003, 2004). Higher DHPG-induced SOCE upon HAP1A overexpression was observed in YAC128 MSNs but not in wildtype cultures, which corresponded to the increase in IP_3_R1 activation in YAC128 MSN cultures that overexpressed HAP1A. The effect of HAP1 protein on the regulation of SOCE in the present HD model was confirmed by HAP1 silencing in YAC128 MSNs that exhibited a reduction of SOCE. This may support the conclusion that HAP1A plays an important role in increasing the activity of IP_3_R1 in the presence of mutHTT. The reduction of HAP1 levels in YAC128 MSN cultures decreased DHPG-induced SOCE through a decrease in the release of Ca^2+^ from the ER to the cytosol. Interestingly, the novel SOC channel inhibitor C_20_H_22_BrClN_2_ that was identified in our previous study (Czeredys et al., 2017) restored SOCE in YAC128 MSNs that overexpressed HAP1A. We also confirmed that higher SOCE in YAC128 MSNs that overexpressed HAP1A was caused by enhanced IP_3_R1 activation.

Among SOC channels, TRPC1 was shown to be involved in SOCE dysregulation and HD pathology in the YAC128 mouse model (Wu et al., 2016, 2018). PLC-mediated PI(4,5)P2 hydrolysis upon the DHPG-induced activation of mGluR1/5 may activate TRPC channels by store-independent ways with diacylglycerol (DAG; Itsuki et al., 2014). We applied a specific inhibitor of IP_3_R1 (Iwasaki et al., 2002; Uchiyama et al., 2002) to confirm that SOCE, which was elevated in the HD model upon HAP1A overexpression, depended on the greater activation of IP_3_R1. When we applied this inhibitor, the SOCE was restored to the normal level in YAC128 MSNs that overexpressed HAP1A, thus refuting the possibility that in YAC128 MSNs that overexpress HAP1A, store-independent TRPC channels are activated depending on PLC-mediated PI(4,5)P2 hydrolysis. Our results suggest that HAP1A may be a new target for pharmacological modulators of SOC that is elevated in HD.

### HAP1A and Its Possible Role in the Regulation of SOCE Machinery in HD

We found that HAP1A increased the activation of IP_3_R1 in YAC128 MSNs that overexpressed mutHTT. The single-cell Ca^2+^ measurements in our cellular model of HD that overexpressed HAP1A showed that the elevation of basal Ca^2+^ levels can be caused by IP_3_R1 hyperactivity. The effect of HAP1 protein expression on higher basal Ca^2+^ levels was also found in MSNs that were transfected with HTT-23Q and HTT-82Q compared with enhanced GFP-transfected MSNs (Tang et al., 2004). One important consequence of higher IP_3_R1 activity is a decrease in ER Ca^2+^ content, which was confirmed by ionomycin pool measurements both in primary neuronal culture (Figure 4) and SK-N-SH cells (Figures 9D,E).

Moreover, when IP_3_R1 was activated by DHPG, we observed an increase in Ca^2+^ release from the ER and an increase in DHPG-induced SOCE. The destabilization of ER Ca^2+^ content affects Ca^2+^ sensors (i.e., STIM1/2 proteins) in the ER lumen. STIM2 is responsible for sensing small changes in Ca^2+^ concentrations in the ER in neurons (Gruszczynska-Biegala et al., 2011; Gruszczynska-Biegala and Kuznicki, 2013) but acts as a weaker SOCE activator than STIM1. Considering that small changes in ER Ca^2+^ content that are caused by IP_3_R1 leakage are highly physiologically relevant, STIM2 may play an important role in aberrant Ca^2+^ signaling in neurodegenerative pathologies. STIM2 knockdown was reported to rescue the amplitude of SOCE and attenuate intracellular Ca^2+^ load in a cellular model of Alzheimer’s disease that was caused by the expression of mutant presenilin 1 M146V (Ryazantseva et al., 2013). STIM2 was shown to regulate ER Ca^2+^ levels in neurons by stimulating the spontaneous activity of SOC channels (Gruszczynska-Biegala et al., 2011; Ryazantseva et al., 2013). The present study took advantage of electrophysiological techniques that allowed us to distinguish pre-activated SOC channels. We found that the expression of HAP1A in SK-N-SH HTT138Q GFP resulted in the appearance of constitutive SOCE.

HAP1 may regulate gene expression (Wu and Zhou, 2009). In the present study, we examined the possible influence of HAP1A on the expression of SOCE machinery-related proteins. We found no differences in the expression of STIM1, Orai1 and TRPC1 but observed an increase in STIM2 expression. Therefore, STIM2 may be involved in the activation of DHPG-induced SOCE in YAC128 MSN cultures that overexpress HAP1A. The upregulation of STIM2 and hyperactivity of IP_3_R1 may underlie the constitutive activity of SOC channels in SK-N-SH HTT138Q HAP1A cells. Additionally, the function of STIM2 in SOC channel activation and dendritic spine dysregulation in YAC128 neuron cultures was previously reported (Wu et al., 2016). It was found that STIM2 protein levels were increased in a mouse model of HD and in samples from HD patients. However, in 3-week-old MSN cultures from YAC128 mice, we found no changes in the levels of mRNA that encodes STIM2 protein compared with wildtype MSNs. Consistent with the findings of Miederer (Miederer et al., 2015), we observed approximately five-fold lower expression levels of mRNA that encodes the regulatory STIM2-1 isoform compared with STIM2-2 (Figure 1B).

In summary, we found that the expression of HAP1A in cells with mutHTT led to dramatic changes in Ca^2+^ homeostasis. Some SOC channels became active in the absence of any further stimulus, likely because of STIM2 overexpression in SK-N-SH HTT138Q HAP1A. HAP1A increased basal Ca^2+^ levels in the cytosol, most likely through a decrease in ER Ca^2+^ content as a result of the greater affinity of IP_3_R1 for IP_3_. These results are consistent with data from YAC128 MSN cultures that overexpressed HAP1A, in which we detected a reduction of ER Ca^2+^ content. Moreover, we observed both greater DHPG-induced Ca^2+^ release from the ER and consequent elevations of SOCE.

Despite the high similarity of SOCE machinery in MSNs and SK-N-SH (Wu et al., 2011; Czeredys et al., 2013, 2017; Vigont et al., 2014), various mechanisms of SOCE regulation in different HD cellular models may result in distinct disturbances caused by the HAP1A-mutant HTT-IP_3_R1 complex. For instance, constitutive SOCE was observed obviously only in SK-N-SH HD model overexpressing HAP1A which can be explained by a different sensitivity of cells to small changes in ER Ca^2+^ content or by different expression levels of STIM proteins. Nevertheless, we have demonstrated that overexpression of HAP1A plays a significant role in the improper calcium homeostasis in HD cellular models. The disjunction of HAP1A-mutant HTT-IP_3_R1 interactions as well as attenuation of pathological STIM2-driven SOCE may impact the development of new drugs for the treatment of HD.

## Author Contributions

MC conceived, designed and performed experiments, analyzed the data and wrote the manuscript. VV designed, performed the experiments, analyzed the data and wrote the manuscript. VB performed the experiments. JK, EK and KM designed the experiments, analyzed the data and wrote the manuscript.

## Conflict of Interest Statement

The authors declare that the research was conducted in the absence of any commercial or financial relationships that could be construed as a potential conflict of interest.
