# Supplementary material for: Huntingtin-Associated Protein 1A Regulates Store-Operated Calcium Entry in Medium Spiny Neurons From Transgenic YAC128 Mice, a Model of Huntington’s Disease
Source: Front Cell Neurosci. 2018 Oct 26;12:381. doi: 10.3389/fncel.2018.00381 (PMC6231533; doi:10.3389/fncel.2018.00381)
Supplement: Supplementary file 1 [file Data_Sheet_1.pdf]

Supplementary methods

Gene Expression Analysis

Gene expression analyses were performed using the 7900HT system (Life Technologies). Gene profiling in the brain structures (i.e. striatum, motor cortex, cerebellum, hippocampus, and cortex) was performed using custom-designed TaqMan low-density arrays (Life Technologies), hereinafter referred to as RT-qPCR arrays. For each array (384 assays), 1.6 μg of cDNA was loaded. The obtained data were analyzed using the relative quantification method and $2^{-\Delta CT}$ formula ($\Delta CT = CT_{target} - CT_{Gapdh}$, where CT denotes the cycle threshold). The list of genes and their annotations included in the RT-qPCR arrays is shown in Table 1 (Czeredys et al. 2013). For RT-qPCR arrays, TaqMan chemistry (4369016, Life Technologies) was used.

Literature:

Czeredys, M., Gruszczynska-Biegala, J., Schacht, T., Methner, A., and Kuznicki, J. (2013). Expression of genes encoding the calcium signalosome in cellular and transgenic models of Huntington's disease. *Front Mol Neurosci* 6, 42.

Figure legends:

Fig. 1 Supplementary data
Relative mRNA level of *Hap1* in different brain regions in YAC128 and wildtype mice measured using custom-made TaqMan low-density PCR arrays.
Relative mRNA levels of *Hap1* in the striatum, motor cortex and cerebellum from 3-month-old YAC128 mice **(A)**. Relative mRNA level of *Hap1* in the striatum, motor cortex, cerebellum, hippocampus and cortex from 3-month-old wildtype mice **(B)**. *$p < 0.05$, **$p < 0.01$. The results were obtained from three independent YAC128 and wildtype mice.

Fig. 2 Supplementary data
Relative mRNA level of *Itpr* isoforms in the striatum of YAC128 measured using custom-made TaqMan low-density PCR arrays.
Relative mRNA levels of *Itpr1*, *Itpr2*, and *Itpr3* in the striatum from 3-month-old YAC128 mice **(A)**. Relative mRNA level of *Itpr1* in the striatum and cerebellum from 3-month-old wildtype mice **(B)**. **$p < 0.01$, ***$p < 0.001$. The results were obtained from three independent YAC128 mice.
