# Supplementary figures and images for: Huntingtin-Associated Protein 1A Regulates Store-Operated Calcium Entry in Medium Spiny Neurons From Transgenic YAC128 Mice, a Model of Huntington’s Disease
Source: Front Cell Neurosci. 2018 Oct 26;12:381. doi: 10.3389/fncel.2018.00381 (PMC6231533; doi:10.3389/fncel.2018.00381)

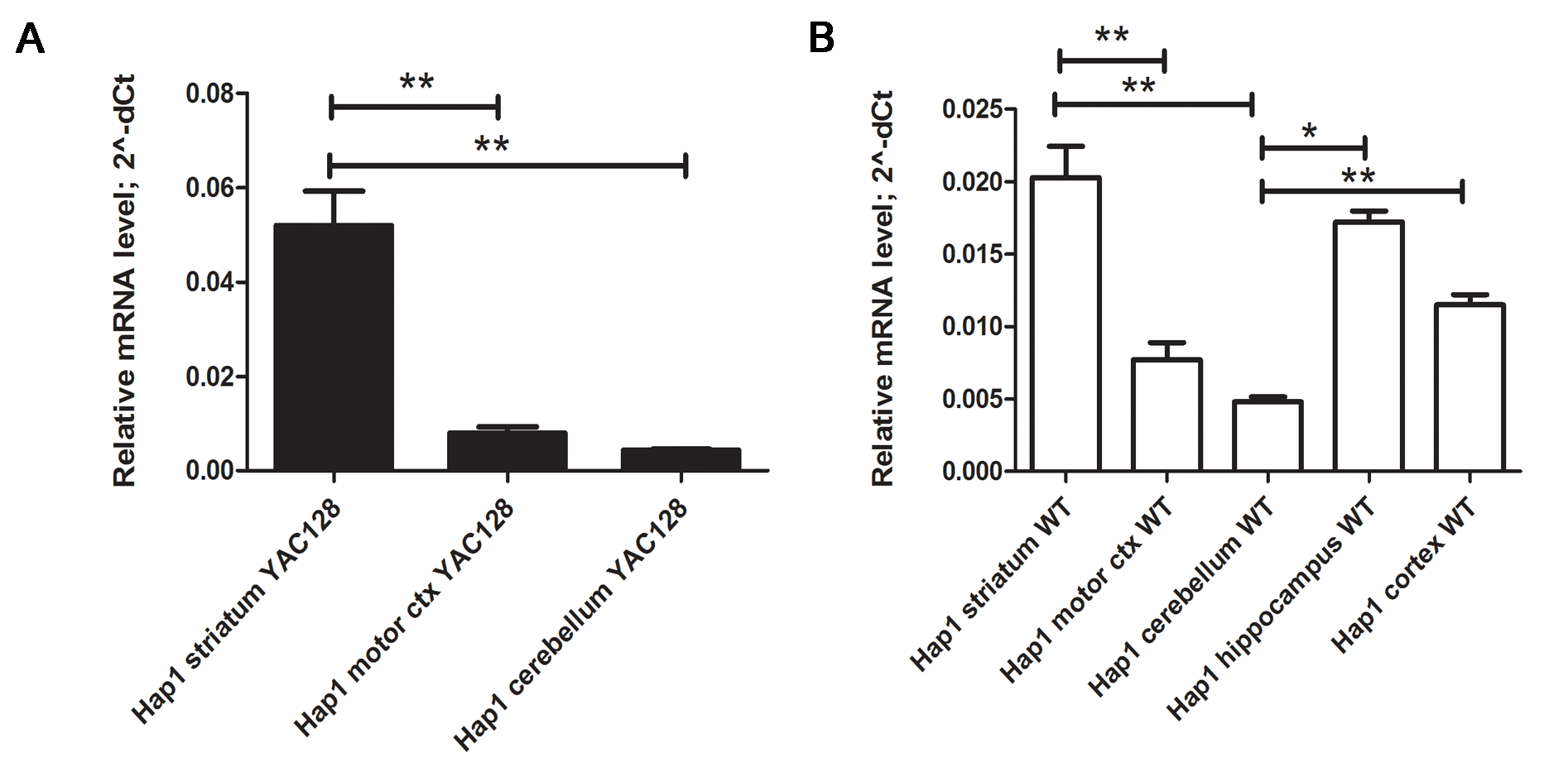

Supplement: Supplementary file 2 [file Image_1.TIF]

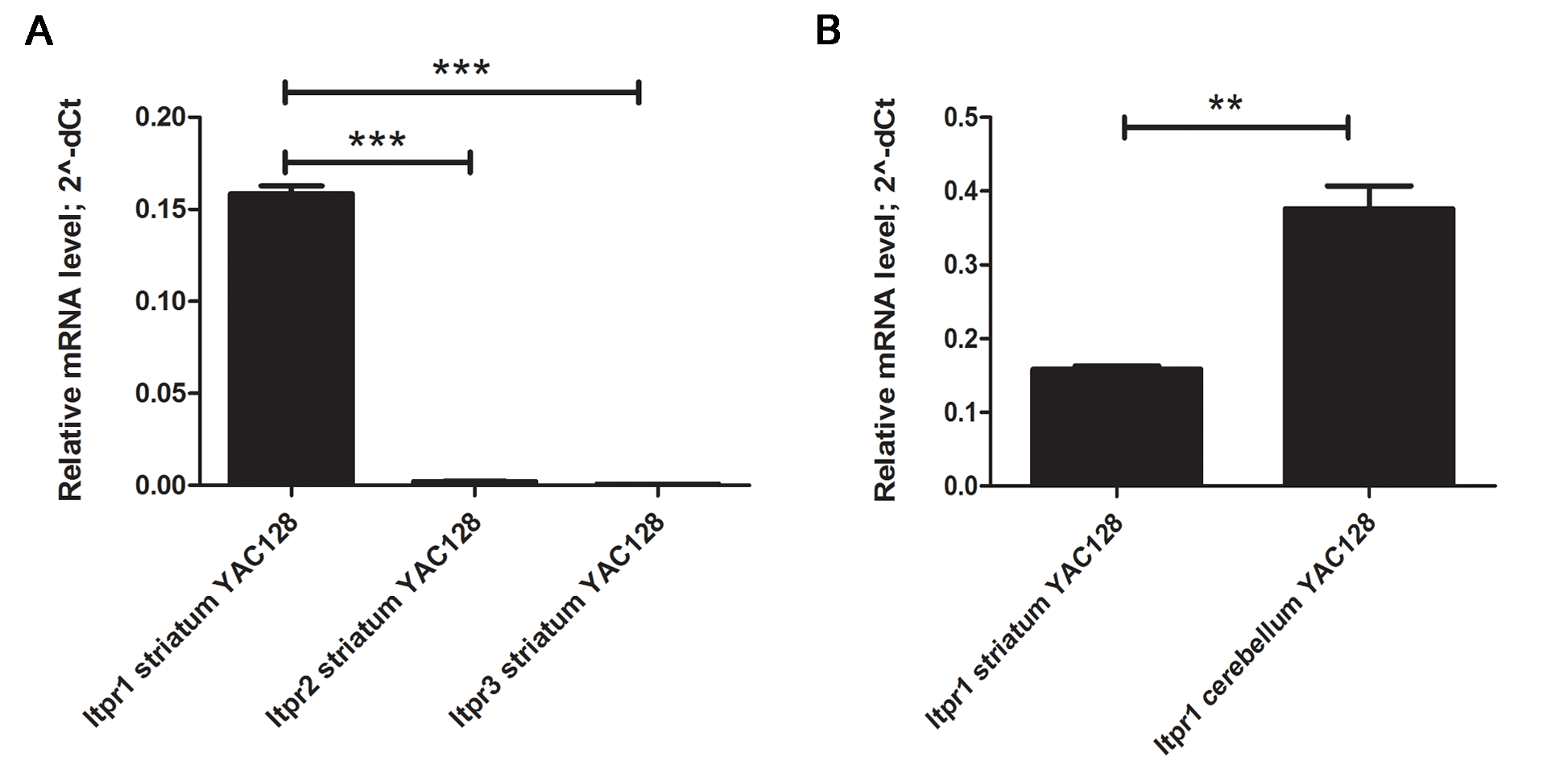

Supplement: Supplementary file 3 [file Image_2.TIF]
